# Association of changes in self-reported sleep duration with mild cognitive impairment in the elderly: a longitudinal study

**DOI:** 10.18632/aging.203149

**Published:** 2021-06-06

**Authors:** Xiyan Wang, Yan Chen, Bixuan Yue, Sifan Li, Qitong Liu, Qiaoyu Li, Lin Li, Jiangwei Sun

**Affiliations:** 1 Department of Public Health, Shenyang Medical College, Liaoning, China; 2 School of Medical Sciences, Örebro University, Örebro, Sweden; 3 Department of Medical Epidemiology and Biostatistics, Karolinska Institutet, Stockholm, Sweden; 4 Institute of Environmental Medicine, Karolinska Institutet, Stockholm, Sweden

**Keywords:** changes in sleep duration, mild cognitive impairment, longitudinal study, China

## Abstract

As a symptomatic predementia stage with progressive cognitive decline, mild cognitive impairment (MCI) is common with aging. How changes in self-reported sleep duration affect MCI risk in the older adults remains unclear. Participants aged ≥ 65 years and enrolled at least two waves in the Chinese Longitudinal Healthy Longevity Survey were included in present longitudinal study. Changes in sleep duration were calculated as the difference between two waves and categorized into five groups: decreased >2 h, decreased 0-2h, stable, increased 0-2 h, and increased >2 h. MCI was measured by the Chinese version of the Mini-Mental State Examination. Generalized estimating equation model and restricted cubic spline function was applied to investigate the association. Among 9,005 participants (mean age, 81.19 years; 4,391 male), 2,877 developed MCI. Comparing with individuals with stable sleep duration, MCI risk [odds ratio (95% confidence intervals)] was: 1.15 (0.99-1.34) for decreased >2 h, 0.99 (0.87-1.13) for decreased 0-2h, 1.09 (0.95-1.24) for increased 0-2 h, and 1.57 (1.36-1.81) for increased >2 h, respectively. Similar patterns were observed among subgroup analyses by sex, age, and sleep quality at baseline. For participants with long sleep duration at baseline (>8h), further increased >2 h was associated with higher MCI risk [2.23 (1.55-3.21)]. Either in the whole or subgroup population, a U-shaped association was observed (*P*_non-linearity_<0.05). In conclusion, changes in self-reported sleep duration were associated with MCI risk in a U-shaped pattern. Strategies that shifting sleep duration into normal range and keeping it stable are essential to prevent MCI in clinical practice.

## INTRODUCTION

Data from the National Bureau of Statistics show that the population aged 65 and above in China had increased to 190.6 million, accounting for 13.5 percent of the total population by the October, 2020 [1]. Health problems such as neurodegenerative diseases are increasing with aging. Dementia, an incurable progressive neurodegenerative disease which results into deterioration in cognition and independent functional abilities, is a leading cause of disability in the elderly [2, 3], which causes severe distress for patients and caregivers and huge public health burden due to spiraling costs [4–6]. The number of individuals with dementia in China is estimated to be 10 million, accounting for approximately 25% of the global patient with dementia [7, 8].

Mild cognitive impairment (MCI), the symptomatic predementia phase of the trajectory of cognitive decline, is a clinical stage between normal aging and dementia, with a 10 to 15 annual conversion rate to dementia [9–11]. It is characterized by a greater cognitive decline rate than expected based on age and educational level, but without significant impairment in independent functional abilities and in social or occupational functioning [12]. Given no medications have proven effective for reversing dementia progression [13, 14], exploring risk factors of MCI is essential for preventing and improving early detection of MCI in clinical practice, particularly in patients with high risk of developing dementia.

The National Sleep Foundation in the United States suggests seven to eight hours as the optimal level of sleep duration for the older adults [15]. Either short or long sleep duration has been found to be associated with a series of adverse health outcomes including stroke, diabetes, cardiovascular disease, and all-cause mortality [16, 17]. Non-optimal sleep duration was also linked to an elevated risk of cognitive impairment [18, 19]. However, there remains some knowledge gaps. First, the oldest-old population (aged over 80 years) were rarely enrolled in previous studies [20, 21], especially those aged more than 90 or 100 years. Second, previous studies are mainly focused on the health effect of sleep duration which was measured at baseline, the influence of changes in sleep duration however was rarely explored. Third, the dose-response relationship between changes in sleep duration and risk of MCI has not been fully studied [22, 23].

Using data from the Chinese Longitudinal Healthy Longevity Survey (CLHLS), we hereby investigated the relationship between changes in self-reported sleep duration and risk of MCI among the elderly adult in China.

## RESULTS

A total of 9,005 participants (mean age, 81.19 years; 4,391 male) were enrolled in present study, and 2,877 participants developed MCI during a median of 6.16 years follow-up (interquartile range: 4.22-9.46 years). Compared with participants with stable sleep duration, those with decreased or increased > 2h were more likely to be older, female, living in rural area, farmer or manual workers and illiterate. They also tend to have lower level of activities in daily living (ADL) score, physical performance score, food diversity score and social activity score. Unlike participants with decreased sleep time, participants with increased sleep time tend to have lower sleep quality and shorter sleep duration at baseline (Table 1).

**Table 1:** Baseline characteristics of the elderly adults, a longitudinal study in China, 2005 to 2019.

		**Changes in self-reported sleep duration**					
**Variables**	**Whole population (n=9,005)**	**Decreased >2 h (n=1,322)**	**Decreased 0-2 h (n=2,271)**	**Stable (n=1,735)**	**Increased 0-2 h (n=2,270)**	**Increased >2 h (n=1,407)**	**P value**
Age at baseline, years							
Mean ± SD	81.19 ± 10.71	83.29 ± 10.49	80.44 ± 10.68	79.70 ± 10.63	80.21 ± 10.47	83.84 ± 10.66	<0.0001
Median (IQR)	80.89 (71.47-89.33)	84.16 (74.44-90.60)	80.05 (70.63-88.40)	78.76 (70.01-87.62)	79.84 (70.83-87.76)	84.97 (74.98-91.46)	
<80 years	4218 (46.84)	497 (37.59)	1126 (49.58)	929 (53.54)	1146 (50.48)	520 (36.96)	<0.0001
80-89 years	2682 (29.78)	447 (33.81)	651 (28.67)	469 (27.03)	668 (29.43)	447 (31.77)	
≥90 years	2105 (23.38)	378 (28.59)	494 (21.75)	337 (19.42)	456 (20.09)	440 (31.27)	
Male, n(%)	4391 (48.76)	614 (46.44)	1134 (49.93)	852 (49.11)	1146 (50.48)	645 (45.84)	0.3866
Ethic, Han, n(%)	8513 (94.54)	1262 (95.46)	2154 (94.85)	1623 (93.54)	2132 (93.92)	1342 (95.38)	0.2023
Marriage status, married, n(%)	4768 (52.95)	778 (58.85)	1142 (50.29)	875 (50.43)	1109 (48.85)	864 (61.41)	0.0003
Ever or current smoker, n(%)	3311 (36.80)	455 (34.47)	871 (38.40)	624 (35.99)	864 (38.08)	497 (35.32)	0.7244
Ever or current drinker, n(%)	2997 (33.33)	427 (32.37)	733 (32.35)	575 (33.18)	792 (34.91)	470 (33.48)	0.4023
Ever or current exerciser, n(%)	3718 (41.41)	490 (37.26)	925 (40.78)	753 (43.53)	993 (43.86)	557 (39.76)	0.6602
Enrollment year, n(%)							0.0012
2005	5185 (57.58)	724 (54.77)	1277 (56.23)	997 (57.46)	1355 (59.69)	832 (59.13)	
2008-2009	2908 (32.29)	456 (34.49)	753 (33.16)	555 (31.99)	682 (30.04)	462 (32.84)	
2011-2012	551 (6.12)	64 (4.84)	144 (6.34)	111 (6.40)	151 (6.65)	81 (5.76)	
2014	361 (4.01)	78 (5.90)	97 (4.27)	72 (4.15)	82 (3.61)	32 (2.27)	
Residence, n(%)							0.0123
City	1648 (18.30)	187 (14.15)	404 (17.79)	384 (22.13)	421 (18.55)	252 (17.91)	
Town	1691 (18.78)	248 (18.76)	427 (18.80)	296 (17.06)	417 (18.37)	303 (21.54)	
Rural area	5666 (62.92)	887 (67.10)	1440 (63.41)	1055 (60.81)	1432 (63.08)	852 (60.55)	
Education, n(%)							<0.0001
Illiterate	5191 (57.65)	831 (62.86)	1281 (56.41)	928 (53.49)	1241 (54.67)	910 (64.68)	
Primary school	2860 (31.76)	405 (30.64)	735 (32.36)	582 (33.54)	741 (32.64)	397 (28.22)	
Middle school or above	954 (10.59)	86 (6.51)	255 (11.23)	225 (12.97)	288 (12.69)	100 (7.11)	
Occupation, n(%)							0.0312
Farmer or manual	6685 (74.24)	1067 (80.71)	1716 (75.56)	1204 (69.39)	1608 (70.84)	1090 (77.47)	
Clerical	1203 (13.36)	144 (10.89)	273 (12.02)	281 (16.20)	337 (14.85)	168 (11.94)	
Professional	751 (8.34)	63 (4.77)	188 (8.28)	183 (10.55)	232 (10.22)	85 (6.04)	
Others	366 (4.06)	48 (3.63)	94 (4.14)	67 (3.86)	93 (4.10)	64 (4.55)	
Good sleep quality, n(%)	6087 (67.60)	1050 (79.43)	1712 (75.39)	1217 (70.14)	1434 (63.17)	674 (47.90)	<0.0001
Sleep time at baseline, n(%)							<0.0001
Short (≤ 6h)	1079 (11.98)	4 (0.30)	84 (3.70)	124 (7.15)	345 (15.20)	522 (37.10)	
Moderate (6-8h)	5276 (58.59)	371 (28.06)	1296 (57.07)	1228 (70.78)	1611 (70.97)	770 (54.73)	
Long (> 8h)	2650 (29.43)	947 (71.63)	891 (39.23)	383 (22.07)	314 (13.83)	115 (8.17)	
High ADL score, n(%)	8324 (92.44)	1202 (90.92)	2123 (93.48)	1619 (93.31)	2118 (93.30)	1262 (89.69)	0.0077
Physical performance score, n(%)							<0.0001
5	6201 (68.86)	882 (66.72)	1602 (70.54)	1253 (72.22)	1616 (71.19)	848 (60.27)	
2.5-4.5	2601 (28.88)	400 (30.26)	630 (27.74)	444 (25.59)	616 (27.14)	511 (36.32)	
0-2.5	203 (2.25)	40 (3.03)	39 (1.72)	38 (2.19)	38 (1.67)	48 (3.41)	
Food diversity score, n(%)							<0.0001
6-8	4408 (48.95)	619 (46.82)	1121 (49.36)	916 (52.80)	1131 (49.82)	621 (44.14)	
4-5	2875 (31.93)	440 (33.28)	740 (32.58)	513 (29.57)	745 (32.82)	437 (31.06)	
0-3	1722 (19.12)	263 (19.89)	410 (18.05)	306 (17.64)	394 (17.36)	349 (24.80)	
Social activity score, n(%)							<0.0001
5-8	1432 (15.90)	159 (12.03)	415 (18.27)	299 (17.23)	409 (18.02)	150 (10.66)	
3-4	4290 (47.64)	625 (47.28)	1072 (47.20)	856 (49.34)	1115 (49.12)	622 (44.21)	
0-2	3283 (36.46)	538 (40.70)	784 (34.52)	580 (33.43)	746 (32.86)	635 (45.13)	
Chronic disease score, n(%)							0.0883
0	4750 (52.75)	743 (56.20)	1215 (53.50)	912 (52.56)	1153 (50.79)	727 (51.67)	
1-2	3609 (40.08)	496 (37.52)	908 (39.98)	689 (39.71)	948 (41.76)	568 (40.37)	
≥3	646 (7.17)	83 (6.28)	148 (6.52)	134 (7.72)	169 (7.44)	112 (7.96)	

Abbreviations: ADL: activities of daily living; IQR, interquartile range; SD standard deviation.

**Figure 1:**
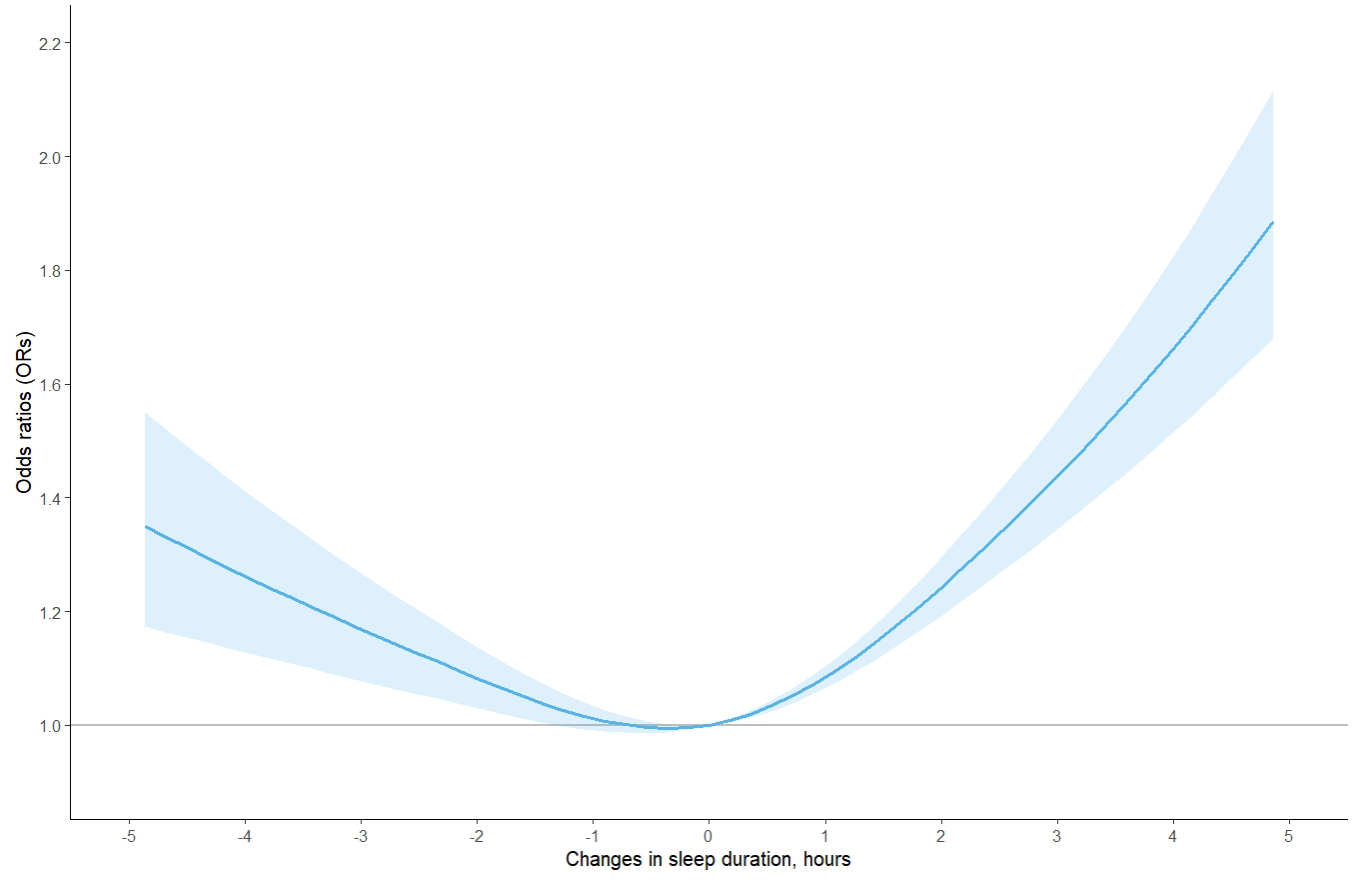
**The adjusted dose-response association between changes in self-reported sleep duration and risk of mild cognitive impairment among the whole population, based on model 4.** Changes in self-reported sleep duration was modeled using a restricted cubic spline function with knots at -2, 0, 2 hours. The reference value was set at zero.

**Figure 2:**
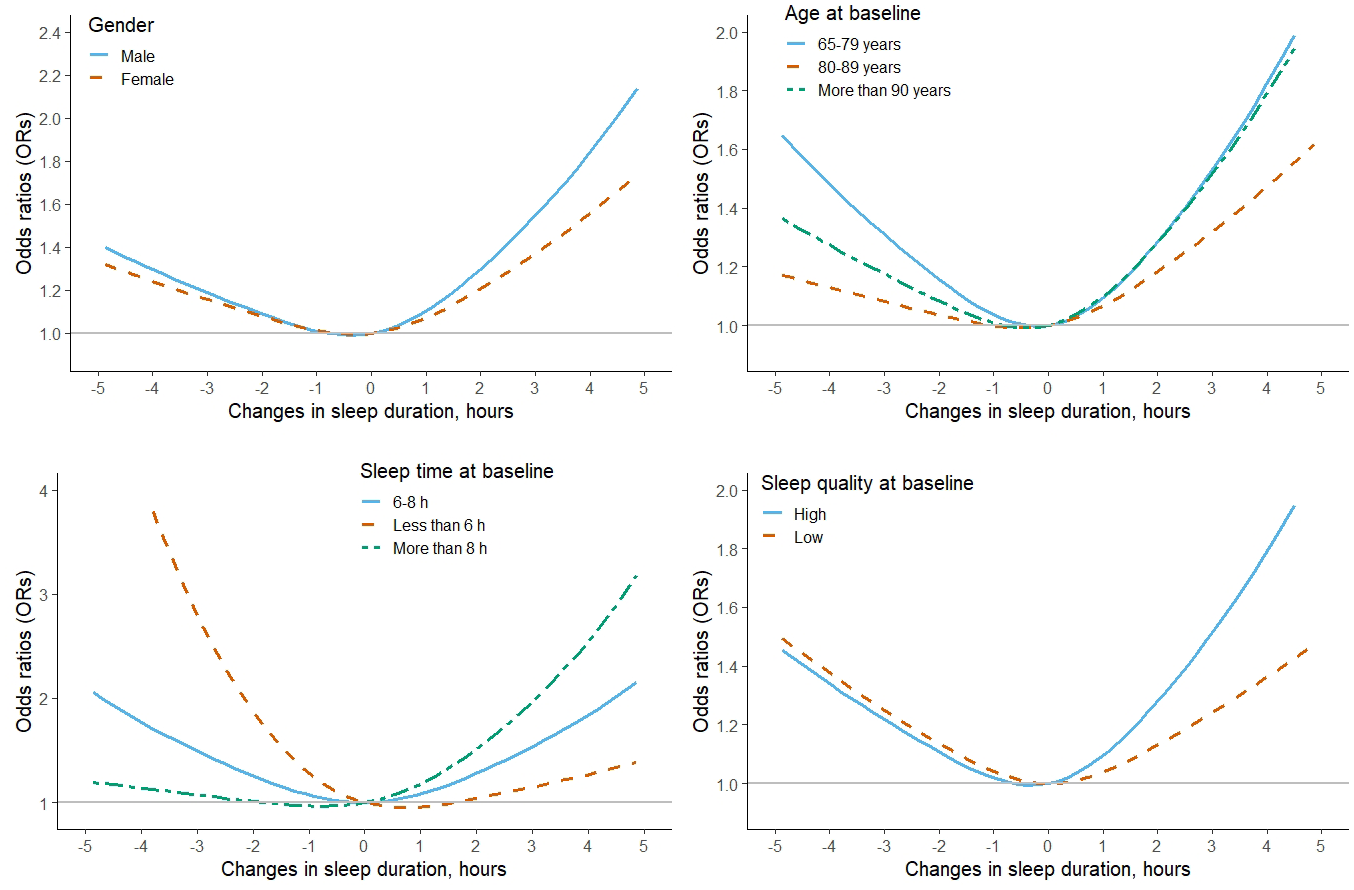
**The adjusted dose-response association between changes in self-reported sleep duration and risk of mild cognitive impairment among the subgroup population, stratified by gender, age, sleep time and sleep quality at baseline, based on model 4.** Changes in self-reported sleep duration was modeled using a restricted cubic spline function with knots at -2, 0, 2 hours. The reference value was set at zero.

In the whole population, a U-shaped association between changes in self-reported sleep duration and risk of MCI was observed via the restricted cubic spline function (Figure 1). The fully adjusted models suggested that compared with individuals with stable sleep duration, the risk of MCI [odds ratio (95% confidence interval): OR (95% CI)] was 1.15 (0.99-1.34) for those with decreased >2 h, 0.99 (0.87-1.13) for decreased 0-2h, 1.09 (0.95-1.24) for increased 0-2 h, and 1.57 (1.36-1.81) for increased >2 h, respectively (Table 2). In the subgroup analyses by sex, age, similar U-shaped patterns were also observed (Figure 2 and Supplementary Table 1).

**Table 2:** Association of changes in self-reported sleep duration with mild cognitive impairment in the elderly, a longitudinal study in China, 2005 to 2019.

			**OR (95% CIs)**			
**Population**	**Groups**		**Model 1**	**Model 2**	**Model 3**	**Model 4**
Whole population						
	Decreased >2 h		1.25 (1.09-1.44)	1.26 (1.10-1.45)	1.25 (1.09-1.45)	1.15 (0.99-1.34)
	Decreased 0-2 h		1.02 (0.90-1.15)	1.02 (0.90-1.15)	1.02 (0.89-1.16)	0.99 (0.87-1.13)
	Stable		1 (Ref.)	1 (Ref.)	1 (Ref.)	1 (Ref.)
	Increased 0-2 h		1.04 (0.92-1.18)	1.04 (0.92-1.18)	1.05 (0.92-1.19)	1.09 (0.95-1.24)
	Increased >2 h		1.48 (1.30-1.68)	1.48 (1.30-1.70)	1.43 (1.25-1.64)	1.57 (1.36-1.81)

Abbreviations: CIs: confident intervals; OR: odds ratio.

Model 1: adjusted for age, sex, and enrollment year; Model 2: model 1 + further adjusted for province, residence, ethic, marriage status, occupation, education; Model 3: model 2 + further adjusted for ADL score, physical performance score, food diversity score, social activity score, and chronic disease score; Model 4: model 3 + further adjusted for sleep time and sleep quality at baseline.

For participants with long sleep time at baseline (>8h), the risk of MCI was sharply increased with the increasing of the changes in sleep time. Among them, further increased >2 h was associated with higher risk of MCI [2.23 (1.55-3.21)], while slight decreasing the sleep time (decreased 0-2h) was related with decreased risk of MCI [0.73 (0.58-0.92)]. However, for participants with short sleep time at baseline (≤6 h), the risk of MCI was gradually decreased with the increasing of the changes in sleep time, although none of the associations were statistically significant (Figure 2 and Supplementary Table 1). The risk of MCI with increased 2h was 1.77 (1.46-2.14) and 1.42 (1.13-1.79) for participants with high or low sleep quality at baseline, respectively (Figure 2 and Supplementary Table 1). The tests for non-linearity in the whole and subgroup population were all less than 0.05 in the restricted cubic spline function.

## DISCUSSION

In the longitudinal study, we observed a U-shaped association between changes in self-reported sleep duration and risk of mild cognitive impairment among the elderly adults in China (*P*_non-linearity_<0.05), after taking into account potential confounders. Robust results were observed in a series of subgroup analyses by sex, age, sleep duration and sleep quality at baseline. We also found that shifting sleep time to a normal range, i.e. from either short or long duration to moderate duration, was related to a decreased risk of MCI.

Few studies have investigated the relationship between changes in sleep duration and MCI risk. One previous prospective study on 7,444 community-dwelling women (65-80 years) found a V-shaped association of habitual sleep duration with both a significant decline in the overall cognitive function and a higher consequent risk of MCI and dementia [24]. Another study among the older Mexican people (mean age: 62.12 years) suggested that increased sleep duration in follow-up period among individuals with moderate sleep duration at baseline (6-9 hours/ night) had a greater decline rate in the overall cognitive function [25]. A recent study using data from CLHLS reported similar associations between sleep duration and MCI as well, but the effect size was smaller in magnitude [26], which might be caused by shorter follow-up time and smaller sample size. An important limitation of the above study is that it did not take the repeated measurement of exposure into consideration, which might be not thoroughly assess the relationship between changes in sleep duration and MCI risk.

In our study, we found that extension of sleep duration by over two hours seems to affect cognitive decline more strongly than other changing groups (OR: 1.57), and those with long sleep duration at baseline (>8h) that further increased over two hours have a greater elevated MCI risk (OR: 2.23). These results indicated that long sleep duration at baseline might also be a potential risk factor for cognitive decline. This argument was supported by the evidence from a review [27], which reported 1.58-times higher risk of poor cognitive function in the self-reported long sleep duration group. Therefore, physicians should be cautious when prescribing psychotropics [28, 29] or hypnotics [30] to the older adults with long sleep duration as these two medications have deterioration effects on cognitive performance as well.

We also found that for participants who sleep less than 6 hours at baseline, MCI risk was gradually reduced when sleep time increased and shifted into a moderate sleep duration. While for those who sleep more than 8 hours at baseline, the risk of MCI was gradually reduced with a reduction in sleep duration by 1-2 hours to moderate sleep duration. These findings were consistent with the recommendation of the National Sleep Foundation, which suggested seven to eight sleep hours for the older adults [15]. Compared with individuals with non-optimal sleep duration, individuals with an optimal one have better cognitive performance, lower incidence of disease, and higher quality of life [24, 31, 32].

Although the mechanisms of the association between changes in sleep duration and risk of MCI remain unclear, several interpretations have been proposed. One is that sleep duration shifting may affect the level of cytokines, such as C-reactive protein (CRP) and interleukin-6 (IL-6) [33, 34], which have a role in regulating inflammatory process, while elevated levels of inflammatory cytokines contribute to the cognitive function decline [35]. Besides, extracellular amyloid-β peptides (a pathological marker of dementia) starts to accumulate in the brain prior to clinical symptoms by years, and its accumulation could cause sleep reduction and fragmentation [36]; while prolonged sleep deprivation may also in turn increase the burden of amyloid-β in the brain [36, 37]. Such evidence supports a bidirectional connection between changes in sleep duration and cognitive function. Moreover, melatonin, having a role on synchronizing circadian rhythm and improving sleep duration and quality, is disrupted in patients with MCI [38], and abnormalities in sleep duration may disturb the circadian rhythm and then prompt the development of MCI [39, 40].

Our results extend previous findings in three aspects. First, the large-scaled population-based longitudinal study provided abundant information and enabled us to conduct a series of subgroup analyses to test the robustness of the results. Second, the enrolled participants were mainly oldest-old adults, among whom the relationship between changes in self-reported sleep duration and cognitive function was rarely studied. Third, the generalized estimating equation model enabled us to address the dependence of records within one participant to obtain an unbiased result, and the restricted cubic spline function further provided clear and visual information of the dose-response associations between changes of self-reported sleep duration and MCI.

There are limitations in present study as well. First, the sleep duration was assessed by self-reported questionnaire, which could overestimate the habitual sleep compared with objectively measured sleep duration and then lead to misclassification of the exposure [41], albeit our results were consistent with those from studies using objectively measurement [42]. Second, different nocturnal and daytime sleep patterns might affect the sleep-inflammation processes (e.g., elevated CRP level) [43], which has been suggested to be associated with the cognitive impairment [35]. However, self-reported measurement precluded the possibility of distinguishing nocturnal from daytime sleep duration. Third, as a screening tool for identifying dementia and psychiatric disorders in an inpatient setting, MMSE however has a ceiling effect, which may be insensitive for detecting early-stage dementia or MCI [44]. Although age, education status and comorbidity (measured by chronic disease score) were carefully considered in our study, those factors could affect its assessment as well. For example, number of comorbidities that affecting cognitive performance increase with advancing age, including sensory impairments, functional disabilities, depression, and vitamin B12/folate deficiency [13], which could further decrease the specificity of MMSE of detecting MCI in a sample like the one included in this study. Therefore, other screening tool that was developed specifically for detection of MCI (e.g., the Montreal Cognitive Assessment [45]) or neuropsychological testing should be considered in practice to detect even subtle cognitive decline among the older adults in future studies. Fourth, although a number of potential confounders were adjusted for in the models and the results were robust across subgroup analyses, residual or unmeasured confounding from other diseases (e.g., depression [46], and sleep-disordered breathing [14, 39]) and medication treatment (e.g., psychotropics [28, 29] and hypnotics [30]) cannot be ruled out. Lacking of such information makes us unable to explore whether the noted associations were independent of those factors. Fifth, the studied population was the elderly Chinese, so the generalization of our findings to other age groups and ethnicities should be carefully considered due to different lifestyles and genetic backgrounds between the studied populations. Finally, the existed diagnosis delay of MCI may cause an inversion of the noted relationship (i.e., reverse causation) [9, 14], the findings therefore were only suggestive and cannot prove causality.

In conclusion, this longitudinal study suggested a U-shaped relationship between changes in self-reported sleep duration and the risk of MCI in the elderly population. Given the impact of sleep duration on cognitive function, maintaining moderate sleep duration may be an important consideration in future clinical practices aimed at alleviating cognitive decline in the elderly population.

## MATERIALS AND METHODS

### Study design

**Figure 3:**
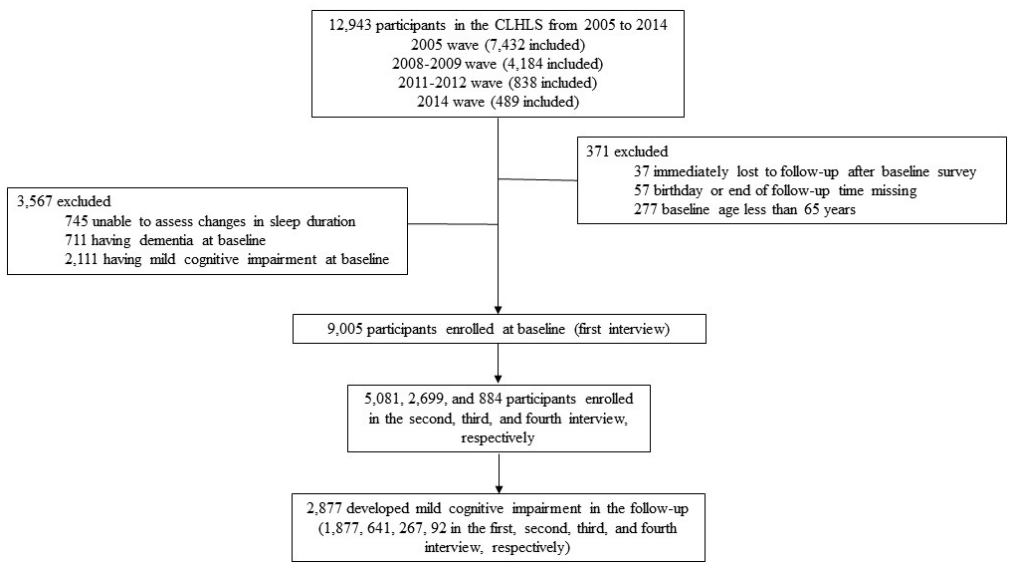
Flow chart of participant selection, a longitudinal study of changes in self-reported sleep duration with risk of mild cognitive impairment among the elderly adults in China, 2005-2019.

The detailed description of CLHLS has been previously published [47]. The CLHLS, established in 1998, was conducted in about half of the cities and counties in 22 of 31 Chinese provinces, and had consequent surveys in 2000, 2002, 2005, 2008-2009, 2011-2012, 2014, and 2018-2019, respectively [48]. Information on basic information, self-evaluation, lifestyle factors, cognitive ability, abilities of performing daily activities, personal background, and family structure of the participants was collected in each wave. Self-reported sleep duration was started to measure from 2005 wave and since then, and due to lacking of follow-up information, participants that newly enrolled in 2018-2019 wave were not considered. Therefore, only participants in the four waves (2005, 2008-2009, 2011-2012 and 2014) were enrolled in the present study. We first excluded 371 subjects from the analysis because of lacking of information on follow-up (n=37), birthday or end of follow-up time (n=57), or younger than 65 years old at baseline (n=277). Then we excluded 3,567 subjects who were unable to assess sleep duration changes (n=745), or had dementia (n=711) or MCI at baseline (n=2,111). As a result, a total of 9,005 participants were finally enrolled, and among them, 5,081, 2,699, and 884 participants were enrolled in the second, third, and fourth interview, respectively. A total of 2,877 participants developed MCI in the follow-up (Figure 3).

### Self-reported sleep duration and quality measurement

Self-reported sleep duration was assessed via question “On average, how many hours do you usually sleep?”. Based on their answers, participants were classified into three categories: short (≤ 6 h), moderate (6-8 h), and long (> 8h) sleep duration. Changes in self-reported sleep duration were calculated as the difference between two waves and categorized into five groups: decreased >2 h, decreased 0-2h, stable, increased 0-2 h, and increased >2 h. Sleep quality was assessed via question “How do you rate your sleep quality?”, and participants were then classified into high or low sleep quality group accordingly.

### MCI measurement

Cognitive performance was measured by the Chinese version of the Mini-Mental State Examination (MMSE), which was one of the most commonly used screening instruments for identifying cognitive impairment. It contains twenty-four items regarding orientation, registration, attention, calculation, recall and language, with a total score ranging from 0 to 30, and higher scores indicate better cognitive function. Because higher education level was associated with better MMSE performance [49], different cut-off points across education levels were applied to define MCI (< 18 for the illiterate, < 21 for those with level of primary school, and < 25 for those with level of middle school or above) [50].

### Statistical analyses

Baseline characteristics were compared across categories of changes in self-reported sleep duration using an analysis of variance for continuous variables and ordinal Chi-square test for categorical variables. Given one participant may have multiple changing status in the follow-up period, the generalized estimating equation model was used to investigate the association via the GENMOD procedure in SAS. Four models that adjusting for different covariates were constructed to test the robustness of the results. In model 1, age, sex and enrollment year (categorical variable: 2005, 2008-2009, 2011-2012, and 2014) was adjusted for. In model 2, province (categorical), residence (city, town and rural area), ethic (Han/others), marriage status [married/others (e.g., widowed, divorced or never married)], occupation (farmer or manual, clerical, professional, and others), and education (illiterate, primary school, and middle school or above) was further adjusted for. Model 3 included covariates in model 2 plus activities in daily living (ADL) score (high/low), physical performance score (categorical: 5, 2.5-4.5, and 0-2.5), food diversity score (categorical: 6-8, 4-5, and 0-3), social activity score (categorical: 5-8, 3-4, and 0-2), and chronic disease score (categorical: 0, 1-2, and ≥3). The definitions of the above scores were described in our previous study [51]. Model 4 was adjusted for the same covariates as model 3 plus sleep time and sleep quality at baseline. A series of subgroup analyses according to gender, age, sleep time and sleep quality at baseline were also conducted to explore whether heterogeneity exists within a population.

Restricted cubic spline function with 3 knots at changing time of “-2, 0, 2 hours” was further applied to explore the dose-response association in the whole and subgroup population. The reference value for changes in self-reported sleep duration was set as zero.

Data analyses and visualization were performed using SAS version 9.4 (SAS Institute Inc, Cary, NC) and ggplot2 package in R version 3.6.0. All statistical tests were 2-sided, and a *P* value less than 0.05 was considered statistically significant.

## Supplementary Material

Supplementary Table 1
