# Supplementary material for: Association of changes in self-reported sleep duration with mild cognitive impairment in the elderly: a longitudinal study
Source: Aging (Albany NY). 2021 Jun 6;13(11):14816–28. doi: 10.18632/aging.203149 (PMC8221318; doi:10.18632/aging.203149)
Supplement: Supplementary Table 1 [file aging-13-203149-s001.pdf]

SUPPLEMENTARY TABLE

Supplementary Table 1. Association of changes in self-reported sleep duration with mild cognitive impairment in the elderly, stratified by age, gender, sleep time and sleep quality at baseline.

Characteristics	Groups	OR (95% CIs)			
		Model 1	Model 2	Model 3	Model 4
Age at baseline, 65-79 years					
	Decreased >2 h	1.52 (1.15-2.01)	1.56 (1.17-2.09)	1.53 (1.14-2.06)	1.34 (0.97-1.84)
	Decreased 0-2 h	1.01 (0.78-1.30)	1.03 (0.79-1.33)	1.04 (0.80-1.35)	0.99 (0.75-1.29)
	Stable	1 (Ref.)	1 (Ref.)	1 (Ref.)	1 (Ref.)
	Increased 0-2 h	0.98 (0.76-1.26)	1.00 (0.77-1.29)	0.99 (0.76-1.28)	1.03 (0.79-1.34)
	Increased >2 h	1.48 (1.12-1.96)	1.54 (1.15-2.05)	1.43 (1.06-1.93)	1.61 (1.17-2.21)
Age at baseline, 80-89 years					
	Decreased >2 h	1.12 (0.90-1.38)	1.13 (0.91-1.40)	1.12 (0.90-1.40)	1.05 (0.83-1.32)
	Decreased 0-2 h	0.99 (0.81-1.20)	0.98 (0.80-1.20)	0.97 (0.79-1.19)	0.95 (0.77-1.17)
	Stable	1 (Ref.)	1 (Ref.)	1 (Ref.)	1 (Ref.)
	Increased 0-2 h	1.05 (0.86-1.28)	1.04 (0.85-1.27)	1.06 (0.86-1.29)	1.09 (0.88-1.34)
	Increased >2 h	1.31 (1.06-1.62)	1.32 (1.07-1.63)	1.27 (1.02-1.58)	1.37 (1.09-1.72)
Age at baseline, ≥ 90 years					
	Decreased >2 h	1.24 (0.99-1.56)	1.24 (0.99-1.57)	1.24 (0.98-1.56)	1.15 (0.90-1.46)
	Decreased 0-2 h	1.03 (0.83-1.27)	1.01 (0.81-1.25)	1.04 (0.84-1.30)	1.01 (0.81-1.26)
	Stable	1 (Ref.)	1 (Ref.)	1 (Ref.)	1 (Ref.)
	Increased 0-2 h	1.07 (0.86-1.32)	1.08 (0.87-1.34)	1.08 (0.87-1.35)	1.12 (0.90-1.40)
	Increased >2 h	1.63 (1.32-2.03)	1.62 (1.30-2.02)	1.62 (1.29-2.03)	1.78 (1.41-2.24)
Male					
	Decreased >2 h	1.46 (1.18-1.80)	1.48 (1.19-1.83)	1.43 (1.14-1.78)	1.32 (1.04-1.66)
	Decreased 0-2 h	1.12 (0.92-1.37)	1.12 (0.92-1.37)	1.10 (0.90-1.36)	1.07 (0.87-1.32)
	Stable	1 (Ref.)	1 (Ref.)	1 (Ref.)	1 (Ref.)
	Increased 0-2 h	1.16 (0.95-1.40)	1.16 (0.96-1.41)	1.18 (0.96-1.44)	1.24 (1.01-1.51)
	Increased >2 h	1.67 (1.36-2.04)	1.70 (1.38-2.09)	1.58 (1.27-1.95)	1.80 (1.43-2.26)
Female					
	Decreased >2 h	1.12 (0.94-1.35)	1.13 (0.94-1.36)	1.14 (0.95-1.38)	1.05 (0.86-1.28)
	Decreased 0-2 h	0.95 (0.81-1.12)	0.94 (0.80-1.11)	0.95 (0.81-1.13)	0.93 (0.78-1.10)
	Stable	1 (Ref.)	1 (Ref.)	1 (Ref.)	1 (Ref.)
	Increased 0-2 h	0.97 (0.82-1.14)	0.97 (0.82-1.14)	0.96 (0.81-1.14)	0.99 (0.83-1.17)
	Increased >2 h	1.36 (1.14-1.61)	1.35 (1.13-1.61)	1.32 (1.10-1.58)	1.41 (1.17-1.70)
Short sleep time at baseline, ≤ 6 h					
	Decreased >2 h	1.57 (0.44-5.59)	1.45 (0.40-5.24)	1.62 (0.44-5.92)	1.65 (0.45-6.01)
	Decreased 0-2 h	1.22 (0.78-1.91)	1.19 (0.75-1.89)	1.11 (0.69-1.77)	1.11 (0.69-1.78)
	Stable	1 (Ref.)	1 (Ref.)	1 (Ref.)	1 (Ref.)
	Increased 0-2 h	0.92 (0.63-1.34)	0.90 (0.61-1.32)	0.87 (0.59-1.28)	0.87 (0.59-1.29)
	Increased >2 h	1.13 (0.79-1.61)	1.08 (0.75-1.56)	0.94 (0.65-1.37)	0.95 (0.65-1.39)
Moderate sleep time at baseline, 6-8 h					
	Decreased >2 h	1.49 (1.19-1.87)	1.53 (1.22-1.93)	1.55 (1.23-1.95)	1.55 (1.23-1.95)
	Decreased 0-2 h	1.17 (0.99-1.39)	1.18 (0.99-1.40)	1.20 (1.01-1.43)	1.20 (1.01-1.43)
	Stable	1 (Ref.)	1 (Ref.)	1 (Ref.)	1 (Ref.)
	Increased 0-2 h	1.12 (0.95-1.32)	1.14 (0.96-1.34)	1.15 (0.97-1.35)	1.15 (0.97-1.35)
	Increased >2 h	1.87 (1.56-2.24)	1.88 (1.57-2.26)	1.83 (1.52-2.21)	1.83 (1.52-2.20)
Long sleep time at baseline, > 8 h					
	Decreased >2 h	0.94 (0.76-1.16)	0.93 (0.75-1.15)	0.92 (0.74-1.14)	0.92 (0.74-1.14)
	Decreased 0-2 h	0.75 (0.60-0.93)	0.73 (0.59-0.92)	0.73 (0.58-0.92)	0.73 (0.58-0.92)
	Stable	1 (Ref.)	1 (Ref.)	1 (Ref.)	1 (Ref.)
	Increased 0-2 h	1.12 (0.86-1.45)	1.10 (0.85-1.43)	1.11 (0.85-1.45)	1.11 (0.85-1.46)
	Increased >2 h	2.17 (1.54-3.06)	2.23 (1.57-3.16)	2.22 (1.54-3.19)	2.23 (1.55-3.21)
High sleep quality at baseline					
	Decreased >2 h	1.24 (1.05-1.47)	1.26 (1.07-1.50)	1.25 (1.05-1.48)	1.17 (0.98-1.40)
	Decreased 0-2 h	0.95 (0.82-1.12)	0.95 (0.81-1.11)	0.94 (0.80-1.11)	0.92 (0.78-1.08)
	Stable	1 (Ref.)	1 (Ref.)	1 (Ref.)	1 (Ref.)
	Increased 0-2 h	1.07 (0.91-1.26)	1.07 (0.91-1.26)	1.08 (0.92-1.28)	1.12 (0.95-1.32)
	Increased >2 h	1.64 (1.37-1.95)	1.64 (1.37-1.96)	1.62 (1.35-1.95)	1.77 (1.46-2.14)

Low sleep quality at baseline					
	Decreased >2 h	1.33 (1.03-1.73)	1.34 (1.02-1.75)	1.28 (0.97-1.68)	1.10 (0.82-1.47)
	Decreased 0-2 h	1.19 (0.95-1.49)	1.19 (0.95-1.50)	1.19 (0.94-1.51)	1.16 (0.91-1.47)
	Stable	1 (Ref.)	1 (Ref.)	1 (Ref.)	1 (Ref.)
	Increased 0-2 h	1.00 (0.81-1.24)	1.01 (0.81-1.25)	1.01 (0.81-1.26)	1.05 (0.84-1.32)
	Increased >2 h	1.37 (1.11-1.69)	1.37 (1.11-1.70)	1.29 (1.04-1.61)	1.42 (1.13-1.79)

Abbreviations: CIs: confident intervals; OR: odds ratio.
Model 1: adjusted for age, sex, and enrollment year; Model 2: model 1 + further adjusted for province, residence, ethic, marriage status, occupation, education; Model 3: model 2 + further adjusted for ADL score, physical performance score, food diversity score, social activity score, and chronic disease score; Model 4: model 3 + further adjusted for sleep time and sleep quality at baseline.
